# Supplementary material for: Environmental properties of cells improve machine learning-based phenotype recognition accuracy
Source: Sci Rep. 2018 Jul 4;8:10085. doi: 10.1038/s41598-018-28482-y (PMC6031649; doi:10.1038/s41598-018-28482-y)
Supplement: Supplementary file 1 — Supplementary information [file 41598_2018_28482_MOESM1_ESM.pdf]

Environmental properties of cells improve machine learning-based phenotype recognition accuracy

Timea Toth[1], Tamas Balassa[1], Norbert Bara[1, 2], Ferenc Kovacs[2], Andras Kriston[1, 2], Csaba Molnar[1], Lajos Haracska[1], Farkas Sukosd[3], Peter Horvath* [1, 4]

Affiliations

[1] Biological Research Centre of the Hungarian Academy of Sciences, Szeged, Hungary.

[2] Single-Cell Technologies Ltd, Szeged, Hungary.

[3] University of Szeged, Department of Pathology, Szeged, Hungary.

[4] Institute for Molecular Medicine Finland (FIMM), University of Helsinki, Helsinki, Finland.

Correspondence and requests for materials should be addressed to P.H. (email: horvath.peter@brc.mta.hu)

Supplementary Note 1

Full list of features

Regular Features: area, ellipse eccentricity, ellipse major axis length, ellipse minor axis length, ellipse orientation, enclosing circle radius, extent, form factor, Haralick texture features (angular second moment, contrast, correlation, sum of squares: variation, inverse difference moment, sum average, sum variance, sum entropy, entropy, difference variance, difference entropy, information measure of correlation), integrated intensity, intensity maximum, intensity mean, intensity median, intensity minimum, intensity standard deviation, perimeter, solidity.

Neighbourhood Features: the minimum, maximum, mean, median, and standard deviation statistics of the regular features; distance features: minimum, maximum, mean, median, standard deviation.

Supplementary Note 2

Significance test

We compare our results to an earlier study, in which the MCF-7 dataset was analysed with Advanced Cell Classifier. In that study, the highest accuracy reached considering regular features only was 88.4%, using the SimpleLogistic (Logistic Boost) classifier, while in this paper we report 90.8% best performance with the combination of regular and neighbourhood features (SMO classifier). The 2.4% difference in accuracy is significant. We randomized our data several times and calculated the accuracies with the mentioned classifiers (Logistic Boost for information from single-cells only, and SMO for data with neighbourhood features).

Advanced Cell Classifier Logistic Boost accuracies:	Neighbourhood SMO accuracies:
88.3443	90.795
88.5236	90.9145
88.2845	90.4363
88.165	90.1973
88.2247	90.9743

As a statistical procedure, two-sample t-test was performed, we considered the result significant at $p < 0.05$.

Descriptive Statistics					
		N	*Mean*	*SD*	*SEM*
ACC		5	88.30842	0.1376	0.06154
Neighbourhood		5	90.66348	0.33386	0.14931
	Difference:		-2.35506		

t-Test Statistics			
	t Statistic	*DF*	*Prob>|t|*
Equal Variance Assumed	-14.58315	8	4.79E-07
Equal Variance NOT Assumed	-14.58315	5.3209	1.71E-05

Null hypothesis: mean1-mean2 = 0
Alternative hypothesis: mean1-mean2 <> 0
At the 0.05 level, the difference is significant.

Supplementary Tables

Table S1. Distribution of the labelled cells in the MCF-7 and the UBC datasets

**MCF-7 Phenotypes**	**Number of labelled cells**	**UBC Phenotypes**	**Number of labelled super-pixels (SLIC 25)**	**Number of labelled super-pixels (SLIC 35)**	**Number of labelled super-pixels (SLIC 50)**	**Number of labelled super-pixels (SLIC 75)**	**Number of labelled super-pixels (SLIC 100)**
Abundant	307	*Cancer cell*	200	200	150	100	75
Rounded	301	*Lumen*	200	200	150	100	77
Bundled microtubule	85	*Endothelial cell*	100	100	75	55	40
Multi-nucleated	155	*Stroma*	200	200	150	100	62
Punctate actin foci	54	*Fibroblast /fibrocyte*	200	200	150	100	62
Decreased cell size	47	*Lymphocyte /Plasma cell*	200	200	150	100	66
Elongated	94	*Smooth muscle*	200	200	150	100	73
Peripheral cytoskeleton	124	*Lipocyte*	200	200	150	100	75
Fragmented nucleus	185	*Debris*	200	200	150	100	68
Debris	321						
**Sum of labelled cells:**	**1673**	**Sum of labelled superpixels:**	**1700**	**1700**	**1275**	**855**	**598**

Supplementary Figures

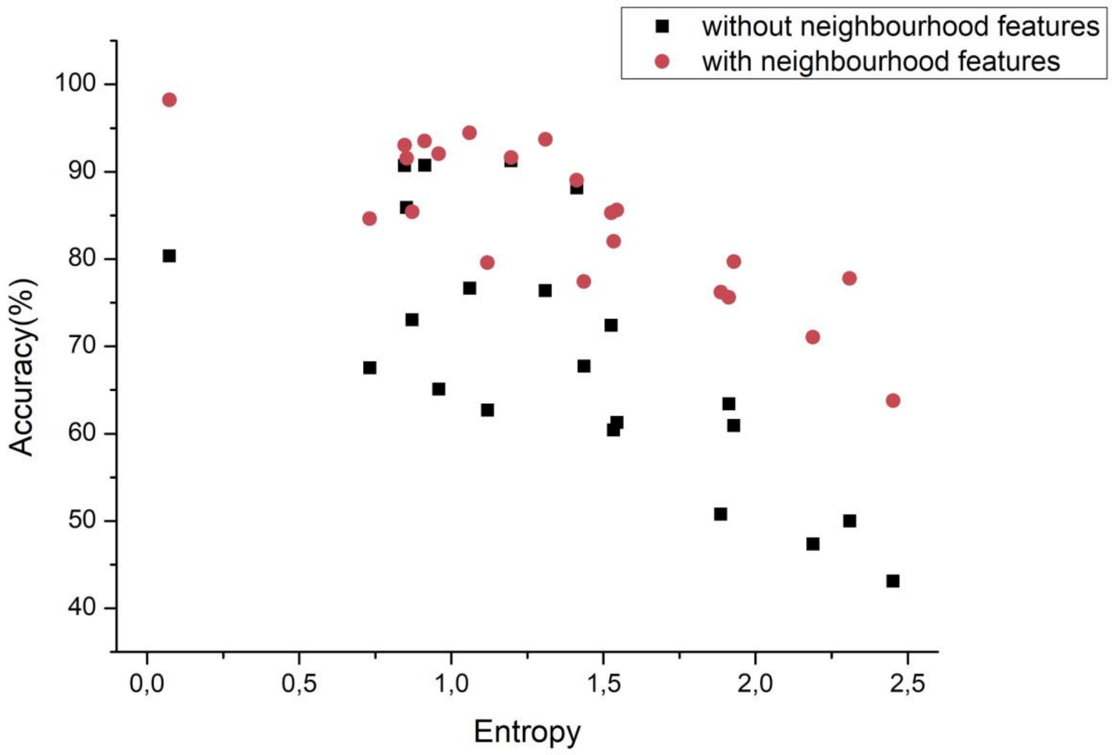

Figure S1. Comparison of the performance of the predictions on different entropy values in the MCF-7 dataset.

Twenty-two images were chosen from the MCF-7 dataset, and all the single cells were annotated on them for ground truth. Entropies of the cells' phenotypes were also calculated for each image. We used the SMO models trained for the whole MCF-7 dataset with regular features only and with neighbourhood features from an 800-pixel-distance (312.2 µm) radius to predict the phenotypes of the cells on the selected 22 images. Predictions were compared to the ground truth to obtain machine-learning accuracies.

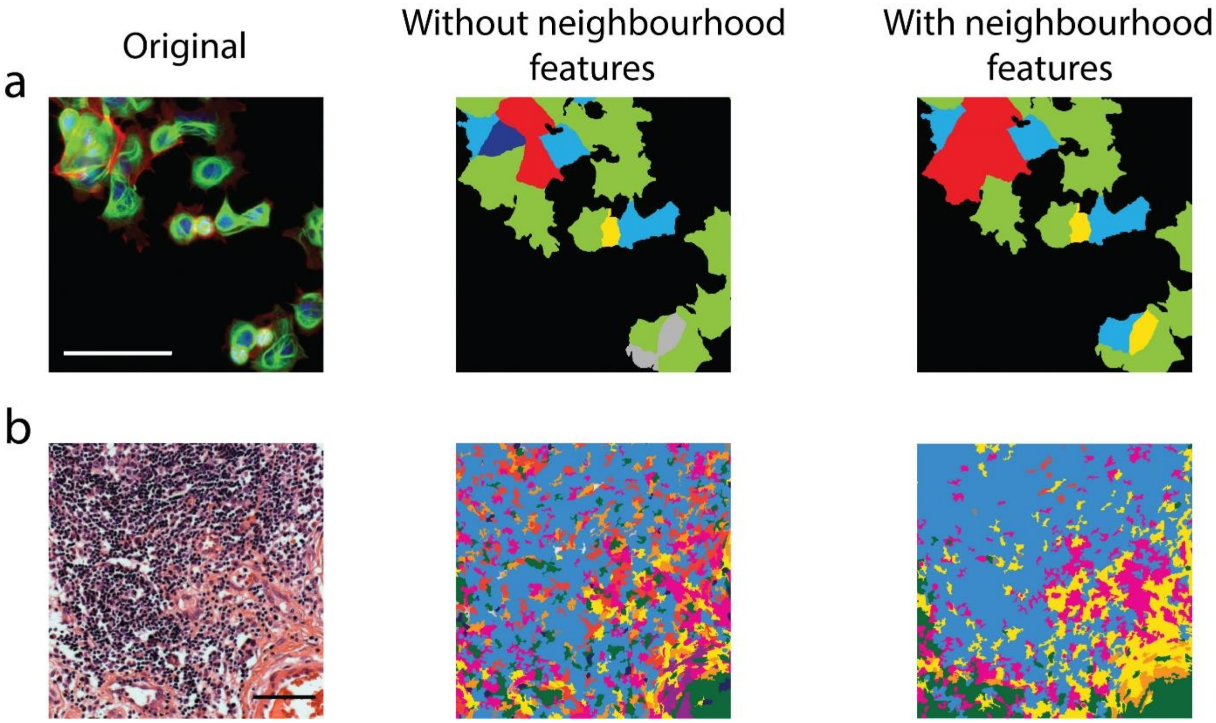

Figure S2. The effect of taking cellular microenvironment into account. **(a)** Prediction examples based on machine learning (SMO) in the cell culture dataset. Original image (left), scale: 50 µm, prediction using regular features only (middle), prediction using regular and neighbourhood features, N-distance: 1200 pixels, 468.3 µm (right). **(b)** Prediction examples based on machine learning (MLP) in the UBC tissue dataset. Original image (left), scale: 50 µm, prediction using regular features only (middle), prediction using the combination of regular and neighbourhood features, KNN, K=100 (right), superpixel size: 35 pixels (9.45 µm)
